# Insights on the practical application of the current treatment consensus in rhabdomyolysis management: a case report

**DOI:** 10.1186/s12882-025-04590-6

**Published:** 2025-11-20

**Authors:** Theresa Gier, Sebastian Koball, Matteo Marcello, Faeq Husain-Syed, Gonzalo Ramírez-Guerrero, Thiago Reis, Steffen Mitzner, Jens-Christian Schewe, Daniel Reuter, Claudio Ronco, Gerd Klinkmann

**Affiliations:** 1 Department of Anaesthesiology, Intensive Care Medicine and Pain Therapy, University Medical Center Rostock, Rostock, Germany; 2 Department of Nephrology, University Medical Center Rostock, Rostock, Germany; 3 International Renal Research Institute of Vicenza (IRRIV), Vicenza, Italy; 4 Department of Nephrology, Dialysis and Kidney Transplantation, San Bortolo Hospital, Vicenza, Italy; 5 Pulmonology and Critical Care Medicine, Member of the German Centre for Lung Research, University Hospital Giessen and Marburg, Giessen, Germany; 6 Nephrology Service, Las Higueras Hospital, Talcahuano, Chile; 7 CPQauali Pesquisa Clinica, Clinical Research Center, São Paulo, Brazil; 8 Division of Nephrology, University of São Paulo, São Paulo, Brazil; 9 Department of Extracorporeal Immunomodulation, Fraunhofer Institute for Cell Therapy and Immunology, Rostock, Germany

**Keywords:** Rhabdomyolysis, CRRT, Hemoadsorption, Case report

## Abstract

**Background:**

Hemoadsorption has emerged as a promising adjunctive therapy in the management of various inflammatory and toxin-mediated conditions. Its role in context of rhabdomyolysis has generated increasing scientific and clinical interest. In response to the recent consensus statement by an international expert task force—published in BMC Nephrology in July 2024—regarding the use of hemoadsorption in rhabdomyolysis, we aim to contribute further to this evolving discourse.

**Case presentation:**

We report on a patient with severe rhabdomyolysis secondary to influenza B infection who suffered acute kidney injury (AKI) as a result. Alongside conservative treatment, we successfully implemented a combined regimen of CRRT and hemoadsorption, while using myoglobin levels up- and downstream of the adsorber as a tool for therapy monitoring. The patient subsequently recovered complete renal function and full body mobility.

**Conclusion:**

The case illustrates the practical implementation of hemoadsorption within a multimodal treatment strategy. In addition to demonstrating concordance with current consensus guidelines, we seek to critically discuss potential refinements in the therapeutic approach—particularly concerning monitoring strategies and the integration of extracorporeal modalities—to facilitate future clinical practice and research.

## Case

A 14-year-old male patient (179 cm, 65 kg) presented to our clinic with severe bilateral groin and thigh pain. His medical history was notable for a febrile respiratory infection one week prior. At admission, no significant pre-existing conditions were identified. Few hours after initial admission to the ward the patient showed signs of progressive clinical deterioration (tachypnea, tachycardia, pronounced mottling of the lower extremities (mottling score 2–3), and a prolonged central capillary refill time of 4–5 s), which led to urgent transfer to the intensive care unit (ICU) with suspected sepsis.

Following microbiological sample collection, empirical antimicrobial therapy with piperacillin/tazobactam and gentamicin was initiated. However, the patient experienced rapid hemodynamic deterioration, exhibiting worsening mottling (score 5), prolonged capillary refill time (8–10 s), tachycardia (130 bpm), tachypnea (35/min), and dyspnea. Echocardiography revealed a reduced biventricular ejection fraction, indicative of cardiogenic shock. Inotropic support with dobutamine (max. 10 µg/kg/min) and milrinone (max. 0.4 µg/kg/min) was commenced.

Given the respiratory distress and escalating hemodynamic instability, the patient underwent endotracheal intubation and mechanical ventilation the same day, followed by advanced hemodynamic monitoring and substantial critical care treatment according to sepsis guidelines. Despite appropriate fluid resuscitation and high doses of catecholamines (incorporating noradrenaline (0.26 µg/kg/min) while substituting dobutamine with adrenaline (0.08 µg/kg/min)) pronounced mottling persisted, alongside a progressive pericardial effusion, suggestive of septic shock with associated septic cardiomyopathy. Additionally, the patient exhibited markedly indurated musculature of the trunk and proximal lower extremities, heightening clinical suspicion of leg compartment syndrome and consecutive rhabdomyolysis. Laboratory findings revealed pronounced systemic inflammation (procalcitonin 6.34 ng/mL, leukocytes 16 × 10⁹/L), metabolic acidosis (pH 7.14, standard bicarbonate 15.3 mmol/L, base excess − 12.7 mmol/L), and hyperlactatemia (5.1 mmol/L), further supporting the diagnosis of septic shock.

## Workup and clinical course

At admission to ICU laboratory analyses confirmed severe rhabdomyolysis (CK 29,269 U/L, myoglobin 26,102 ng/mL), PCR diagnostics of tracheal secretions detected influenza B virus.

Initial management of rhabdomyolysis included escalating the initial fluid supplementation of 2 ml/kg/h to 11 ml/kg/h alongside urine pH monitoring and urine alkalization (9 doses of 8.4 g bicarbonate in the first 48 h). Due to markedly elevated myoglobin levels, continuous renal replacement therapy (CRRT) was initiated. The initial modality was regional-citrate-anticoagulated continuous veno-venous hemodialysis (RCA-CVVHD, using a standard high-flux CRRT hemofilter Fx80, Fresenius Multifiltrate Pro, Fresenius Medical Care, Bad Homburg, Germany) with a blood flow of 100 mL/min, dialysate flow of 2000 mL/h, and citrate/calcium adjustments as required. To enhance myoglobin clearance, a hemoadsorption (CytoSorb^®^ cartridge, Cytosorbents Europe GmbH, Berlin, Germany) was integrated into the extracorporeal circuit in a pre-filter position. Therapy monitoring was optimized by aiming to collect blood samples every six hours from upstream and downstream of the adsorber to evaluate myoglobin clearance. Adsorber replacement was performed when myoglobin concentrations equalized, indicating saturation. The table below (Table 1) shows an overview over myoglobin measurements and adsorber replacements.

**Table 1:** Myoglobin kinetics and adsorber replacement

	Systemic Myoglobin [ng/l]	Pre-Cartridge [ng/l]	Post-Cartridge [ng/l]
− 8 h	26,102		
− 3 h	30,163		
0	Adsorber Implementation		
+ 6 h		47,263	40,339
+ 7 h	Adsorber Replacement		
+ 10 h	44,977		
+ 16 h	66,114		
+ 19 h		76,883	76,270
+ 21 h	Adsorber Replacement		
+ 27 h	72,638		
+ 28 h		70,612	66,071
+ 30 h	Adsorber Replacement		
+ 38 h		67,501	63,290
+ 46 h	57,438		
+ 46 h	Adsorber Removal		

**Fig. 1:**
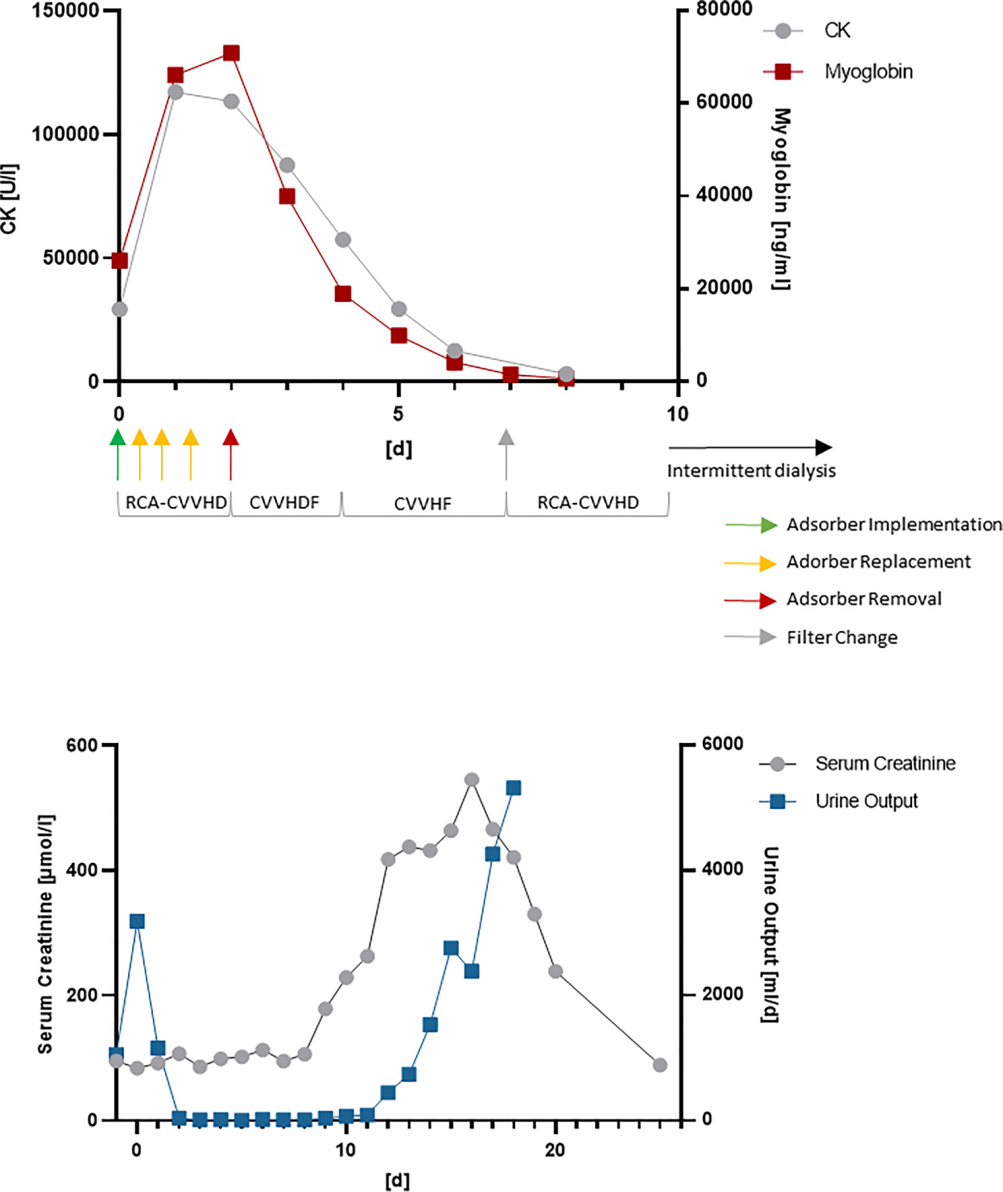
Myoglobin kinetics and Urine Output during ongoing CRRT

Hemoadsorption was discontinued at 46 h as myoglobin kinetics were consistently declining and the prescribed CRRT dose was considered adequate to sustain further clearance. The circuit was then reconfigured to heparin-anticoagulated continuous veno-venous hemodiafiltration (CVVHDF, using a standard high-flux CRRT hemofilter Fx80, Fresenius Multifiltrate Pro, Fresenius Medical Care, Bad Homburg, Germany) to streamline therapy and adjust anticoagulation, using a blood flow of 150 mL/min, a dialysate flow of 4500 mL/h, and a postdilution substitution flow of 1000 mL/h. Two days later, treatment was further intensified to heparin-anticoagulated continuous veno-venous hemofiltration (CVVHF, using a standard high-flux CRRT hemofilter Fx80, Fresenius Multifiltrate Pro, Fresenius Medical Care, Bad Homburg, Germany) in postdilution mode with a substitution flow of 5000 mL/h. The initial preference for RCA-CVVHD and the choice of postdilution flow was based on concerns regarding clotting. A filter change was required after three days due to clot formation, leading to a switch back to standard RCA-CVVHD on day seven. On day eleven after implementation of RRT the patient was switched from CRRT to an intermittent HD regimen, which he was successfully weaned from on day sixteen. The following figure (Fig. 1) presents the kinetics of key monitoring parameters along with the implemented therapy regimen.

During the following weeks on the ICU, the patient exhibited progressive clinical improvement with normalization of renal function and resolution of metabolic disturbances. After 28 days of intensive care treatment, he was discharged to a rehabilitation facility. At follow-up 6 months after discharge from the hospital, he had regained independent mobility, resumed schooling, and demonstrated complete renal recovery.

## Discussion

The management of life-threatening rhabdomyolysis, particularly in severe cases complicated by acute kidney injury (AKI), remains challenging. Forni et al. recently outlined key recommendations for the treatment of rhabdomyolysis, emphasizing early fluid resuscitation—not only to restore volume deficits but also to promote targeted diuresis [1]. Preserving native renal function remains the primary objective, as the glomerulus surpasses any artificial blood purification technique in effectively eliminating pathophysiologically relevant substances such as myoglobin [2]. In cases where renal function proves inadequate despite conservative measures and severe myoglobinuria persists, extracorporeal blood purification should be considered. Our case closely aligns with these recommendations while providing additional insights into optimizing extracorporeal treatment strategies. First, we successfully implemented a multimodal therapeutic approach integrating volume resuscitation and renal replacement therapy (RRT) in conjunction with hemoadsorption, which has also been successfully applied by other groups [3–5]. This strategy is consistent with Forni et al.‘s recommendation that RRT should be initiated in cases of rhabdomyolysis presenting with severe metabolic disturbances or rapidly progressing AKI [1]. A crucial refinement in our approach involved direct monitoring of myoglobin levels upstream and downstream of the adsorber. This allowed for real-time assessment of adsorption efficiency, enabling timely cartridge replacement and optimizing therapy effectiveness. In line with goal-directed strategy, hemoadsorption was stopped once downward myoglobin kinetics were established and CRRT dose sufficiency was achieved, aiming to avoid unnecessary cartridge exposure while maintaining clearance via membrane-based therapy.

Adsorber saturation – evidenced by a plateau of myoglobin decline and convergence of pre-/post-adsorber concentrations (i.e., a falling extraction ratio) – has been widely observed. To mitigate saturation centers have employed (i) time-driven cartridge exchange at fixed intervals or biomarker-guided exchange based on serial myoglobin measurements [4, 5], and, in some reports, (ii) dual-cartridge configurations run in parallel to increase adsorptive capacity and delay breakthrough [6]. Whether parallel hemoadsorption confers superior myoglobin clearance or prolongs effective adsorption compared with sequential single-cartridge strategies requires comparative evaluation in larger cohorts with standardized dosing concepts (effective adsorber hours) and extraction-ratio monitoring.

In our patient, CK concentrations declined in parallel with myoglobin, a finding also described by others [3, 4]. Given that creatine kinase is a ~ 80–86 kDa dimer – exceeding the ~ 60 kDa adsorption cutoff – its reduction is unlikely to reflect cartridge adsorption and more plausibly relates to membrane-based CRRT-clearance and/or endogenous kinetics. Consequently, CK is not a reliable pharmacodynamic marker of hemoadsorber performance. By contrast, myoglobin (~ 17 kDa) is the direct target of adsorption and represents the preferred monitoring analyte for early detection of biochemical saturation and timely cartridge exchange [7].

Furthermore, while Forni et al. discuss the potential benefits of high cut-off (HCO) and medium cut-off (MCO) membranes, further research is needed to delineate their role in rhabdomyolysis management [1]. Integrating hemoadsorption with MCO membranes may help mitigate competition between cytokine and myoglobin clearance, a particularly relevant consideration in viral infection-induced rhabdomyolysis. Consistent with the consensus statement and our case experience, we recommend adjunctive hemoadsorption for patients with persistent severe myoglobinemia despite adequate volume resuscitation, ideally initiated within 24 h and guided by serial myoglobin monitoring. Specifically, we advise pre- and post-adsorber myoglobin measurements approximately every 3 h during the first 12–24 h, with cartridge exchange at biochemical saturation, operationalized as convergence of pre- and post-adsorber concentrations (i.e., loss of a clinically meaningful extraction ratio). Where such monitoring is not feasible, a fixed exchange interval of 8–12 h may be used as a surrogate, in line with consensus practice [1]. Importantly, dosing should be conceived as effective adsorber-hours: three 8-hour cartridges are not equivalent to a single 24-hour cartridge because of saturation dynamics over 6–12 h. Future research should refine coupling strategies with MCO/HCO membranes and optimize blood-flow targets and dialysis modality to maximize myoglobin removal [8].

Another critical aspect of treatment optimization pertains to antimicrobial stewardship. Although gentamicin was included in the initial antimicrobial regimen, its nephrotoxic potential was carefully evaluated and led to its elimination from the therapy regimen as soon as the kidney injury became apparent. Evidence suggests that piperacillin/tazobactam, unlike vancomycin in combination with piperacillin/tazobactam, may offer a more favorable renal safety profile [9]. These considerations mandate judicious antimicrobial selection in critically ill patients at risk of AKI, coupled with close therapeutic drug monitoring (TDM). Moreover, because hemoadsorption can adsorptively remove selected anti-infectives (e.g., vancomycin), regular TDM – with dose and timing adjustements relative to cartridge changes – is essential to ensure target exposure.

The role of bicarbonate administration for urine alkalization remains a matter of debate [10, 11]. While theoretically advantageous in preventing myoglobin precipitation in renal tubules, recent consensus guidelines do not endorse its routine use due to insufficient clinical evidence demonstrating efficacy in AKI prevention. Our case supports this, as renal replacement therapy ultimately became necessary despite initial bicarbonate administration.

Overall, this case report not only demonstrates the successful implementation of current consensus recommendations but also highlights advancements in therapy monitoring through direct myoglobin measurements. While saturation effects of the adsorber cartridge were promptly identified and mitigated, similar considerations should extend to the dialysis membranes utilized in RRT. While filtration-based procedures seem to be less prone to saturation effects and CVVH is superior to CVVHD in terms of myoglobin elimination, comparative studies are needed to further explore the impact of such differences.

In conclusion, these findings underscore the need for further investigation into the timing and configuration of extracorporeal therapy strategies. In particular, optimizing hemoadsorption efficiency and evaluating the role of HCO/MCO membranes remain crucial for developing individualized, evidence-based therapeutic approaches for patients with severe rhabdomyolysis and impaired renal function.

## Data Availability

The datasets used and/or analysed during the current study are available from the corresponding author on reasonable request.

## Abbreviations

CK: Creatine kinase
(C)RRT: (Continuous) Renal replacement therapy
RCA: Regional citrate anticoagulation
CVVHD: Continuous veno-venous hemodialysis
CVVHDF: Continuous veno-venous hemodiafiltration
CVVHF: Continuous veno-venous hemofiltration
HD: Hemodialysis
AKI: Acute kidney injury
HCO: High cutoff membrane
MCO: Medium cutoff membrane
